# Increased blood BACE1 activity as a potential common pathogenic factor of vascular dementia and late onset Alzheimer's disease

**DOI:** 10.1038/s41598-020-72168-3

**Published:** 2020-09-11

**Authors:** Giovanni Zuliani, Alessandro Trentini, Valentina Rosta, Remo Guerrini, Salvatore Pacifico, Stefania Bonazzi, Anna Guiotto, Angelina Passaro, Davide Seripa, Giuseppe Valacchi, Carlo Cervellati

**Affiliations:** 1 Department of Morphology, Surgery, and Medical Sciences, University of Ferrara, Via Luigi Borsari 46, 44121 Ferrara, Italy; 2 Department of Biomedical and Specialist Surgical Sciences, University of Ferrara, Via Luigi Borsari 46, 44121 Ferrara, Italy; 3 Department of Chemical and Pharmaceutical Sciences, University of Ferrara, Via Luigi Borsari 46, 44121 Ferrara, Italy; 4 Department of Medical Sciences, Casa Sollievo della Sofferenza, Viale Cappuccini, 1, 71013 San Giovanni Rotondo, Italy; 5 Animal Science Department, Plants for Human Health Institute, NC State University, 600 Laureate Way, Kannapolis, NC 28081 USA

**Keywords:** Neuroscience, Biomarkers, Diseases

## Abstract

Late onset Alzheimer disease (LOAD) is traditionally considered as a separate disease from vascular dementia (VAD). However, growing evidence suggests that β-amyloid (Aβ) accumulation, that initiates LOAD-related neurodegeneration, is preceded by vascular events. Previous in vitro studies showed that β-secretase 1 (BACE1), the key-enzyme of amyloidogenesis, is upregulated by cerebrovascular insult; moreover, its activity is increased both in brain and serum of LOAD patients. We aimed to investigate whether BACE1 serum activity is altered also in dementias related, or not, to cerebrovascular disease. Thus, we evaluated serum BACE1 activity in a sample of individuals, including patients with LOAD (n. 175), VAD (n. 40), MIXED (LOAD/VAD) dementia (n. 123), other types of dementia (n. 56), and healthy Controls (n. 204). We found that BACE1 was significantly higher not only in LOAD (+ 30%), but also in VAD (+ 35%) and MIXED dementia (+ 22%) (*p* < 0.001 for all), but not in the other types of dementia (+ 10%). Diagnostic accuracy was 77% for LOAD, 83% for VAD, and 77% for MIXED dementia. In conclusion, we showed for the first time that the increase in peripheral BACE1 activity is a common feature of LOAD and VAD, thus underlying a further pathogenic link between these two forms of dementia.

## Introduction

Alzheimer’s disease (AD) and vascular dementia (VAD) are the most common causes of dementia syndrome, with AD accounting for about 70% of cases^1^. Although both pathologies lead to cognitive impairment, AD and VAD have been traditionally considered as two distinct diseases^2^. In fact, if from one side Amyloid-β (Aβ) formation and deposition in neuritic plaques and neurofibrillary tangles (NFT, due to hyperphosphorylation of tau protein) are the main pathophysiological hallmarks of AD, VAD is an heterogeneous group of brain disorders caused by cerebrovascular deterioration, with cortical/subcortical ischemic infarctions and leukoaraiosi^3^.

In contrast with the idea of AD and VAD as different disorders, there is abundant evidence that highlights the involvement of cerebrovascular disease in AD dementia^3–5^. This consideration mostly applies for the late onset form of AD (LOAD), a multifactorial and complex disease where a number of different abnormalities concur to cause the pathophysiological traits^4,6^. In particular, it has been proposed that vascular dysregulation might play an important role in LOAD development, and could contribute to Aβ deposition, functional impairment, and brain atrophy^4,7^. For instance, it has been amply proved that ischemic and neurodegenerative pathology coexist and reciprocally interact in LOAD, impacting the clinical presentation of the disease and it is well-known that LOAD and VAD share important cardiometabolic and lifestyle risk factors^8^.

Despite the convergent experimental and epidemiological evidence supporting the implication of vascular abnormalities in LOAD pathogenesis^3,4,9^, the mechanisms and the cause-effect nature of this interaction is still unclear. The most convincing mechanistic hypotheses are based on the observations that cerebral hypoperfusion may enhance Aβ formation and aggregation^8,10,11^. Increase in Aβ burden caused by vascular factors could occur through the impairment of the clearance (vascular pathway is estimated to be a major route of removal of Aβ from the brain^12^) and/or in the production of the peptide^11^. Relevant to this context, in vivo and in vitro studies showed that hypoperfusion/hypoxia, and induced oxidative stress, may facilitate Aβ production by activating the APP cleavage enzyme β-secretase 1 (BACE1)^13,14^. This is the key-enzyme of the amyloidogenic processing of APP, catalyzing the rate-limiting initial cleavage at the β site of APP; the subsequent cleavage by γ-secretase leads to the generation of Aβ. BACE1 has been recently found to be increased in the brain/CSF(cerebrospinal fluid) of patients with LOAD or mild cognitive impairment (MCI)^15,16^. BACE1 is present also in serum, as recently demonstrated by our group^17^ and Shen et al.^18^ in two recent studies. In particular, we found that LOAD patients has significantly higher levels of serum BACE1 activity compared to controls, and this difference is independent of possible confounders including age, gender, and other risk factors for dementia^17^. Based on this corollary of evidences, in the present study we investigated whether an increase in serum BACE1 activity is a specific feature of LOAD or might also characterize VAD. To this purpose, we evaluated the serum levels of BACE1 in a large sample of elderly individuals (n. 598) including patients affected by LOAD, VAD, mixed LOAD-VAD dementia (MIXED), and other type of dementia, in comparison with cognitively healthy subjects.

## Results

### Demographic and main clinical characteristics of the population sample

In Table 1 are reported the main demographic and clinical characteristics of the subjects included into the study according to diagnosis. Controls were younger (*p* < 0.001 for all *post-hoc* tests) and presented a lower prevalence of female gender (*p* < 0.01) compared to all the other groups; a similar trend was observed for comorbidities (in particular hypertension and cardiovascular disease, CVD). Years of formal education, as well as MMSE, IADL and BADL score were higher in Controls compared with all dementia groups.

**Table 1:** Principal characteristics of the sample according to diagnosis.

Characteristics	Controls (n:204)	Load (n:175)	VAD (n:40)	Mixed (n:123)	Other dementias (n:56)
Age (years)*	74 ± 5	78 ± 6^a^	79 ± 7^a^	80 ± 5^a^	77 ± 6^a^
Female gender (%)*	49	68^a^	73^a^	70^a^	67^a^
Formal Education (years)*	11 (8–13)	5 (3–5)^a^	2 (3–6)^a,b^	5 (4–5)^a^	5 (5–9)^a^
MMSE score (/30)*	27 (26–30)	21 (18–23)^a^	20 (16–23)^a^	20 (16–23)^a^	22 (18–25)^a^
-Current smoker (%)	7	5	12^a^	5	5
**Comorbidities**					
Hypertension (%)*	41	67^a^	79^a^	59^a^	56^a^
Diabetes (%)	8	13	22^a^	17	15
CVD (%)*	5	8	19^a,b^	21^a,b^	15
**Functional status**					
-IADLs*	7 (5–8)	3 (2–6)^a^	4 (2–6)^a^	3 (1–6)^a^	4 (1–7)^a^
-BADLs*	6 (5–6)	6 (5–6)^a^	5 (4–6)^a^	5 (4–6)^a^	5 (4–6)^a^
BACE1 (kU/L)^#^	15 (12–18)	21 (16–27)^a^	23 (18–32)^a^	19 (17–26)^a^	17 (15–20)^b,c,d^

Continuous variables are expressed as mean ± SEM or median (interquartile range). Categorical variable are expressed as percentage within group.

*CVD* cardiovascular diseases, *MMSE* mini mental state examination, *LOAD* late onset Alzheimer’s disease, *IADL* instrumental activities of daily living, *BADL* basic activity of daily living.

*Post-hoc test*: ^a^*p* < 0.05 vs controls; ^b^*p* < 0.05 versus LOAD; ^c^*p* < 0.05 versus VAD; ^d^*p* < 0.05 versus MIXED.

### Serum BACE1activity across the study groups

**Figure 1:**
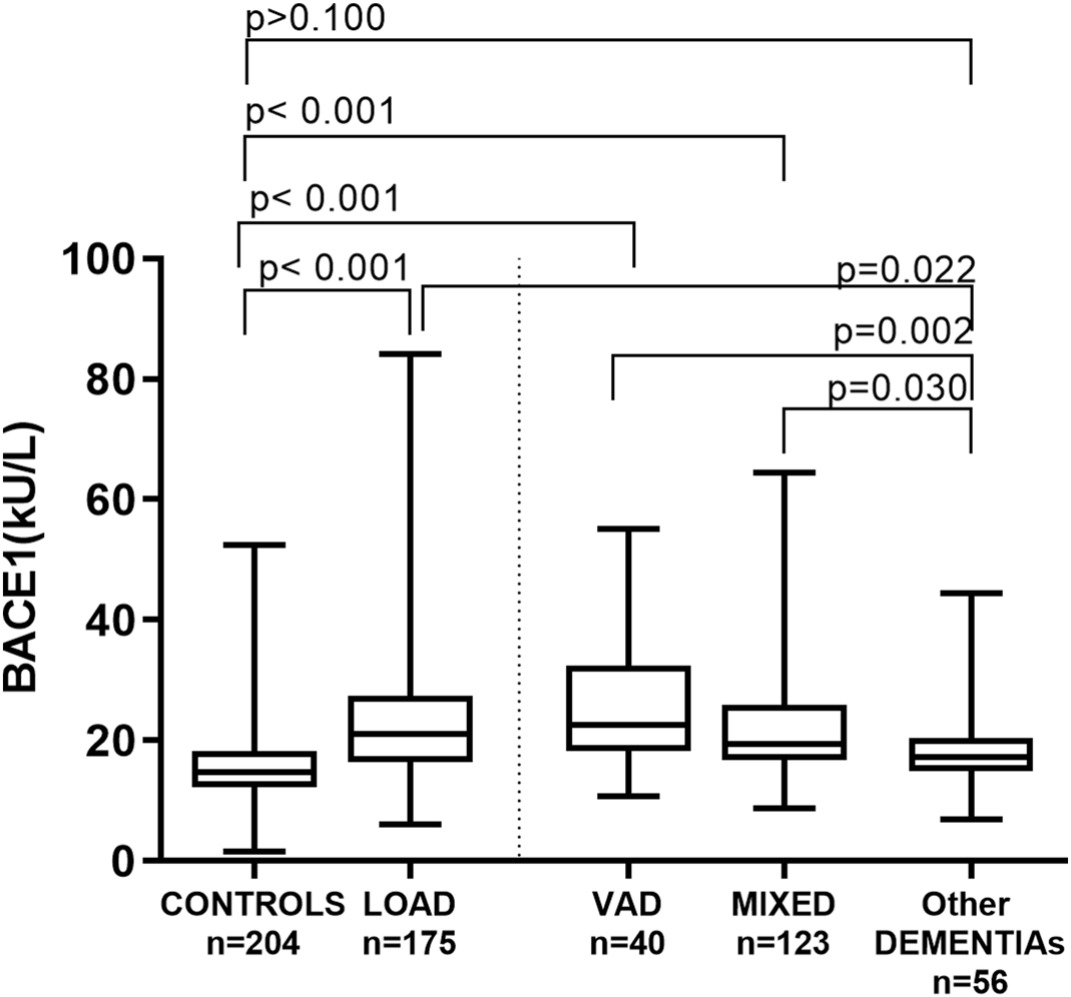
Serum BACE1 activity in Controls, LOAD, VaD, MIXED dementia, and other dementia. Serum BACE1 activity is significantly higher in LOAD (+ 30%), VAD (+ 35%) and MIXED dementia (+ 22%) (*p* < 0.001 for all *post-hoc* comparisons), but not in the Other Dementias group (+ 12%; *p* > 0.10).

As displayed in Fig. 1, compared with Controls, serum BACE1 activity was significantly higher in LOAD (+ 30%), VAD (+ 35%) and MIXED dementia (+ 22%) (*p* < 0.001 for all post-hoc comparisons), but not in the Other Dementias group (+ 12%; *p* > 0.10).

As the second step, we evaluated the possible influence of potential confounding factors on the association between BACE1 activity and the diagnosis of LOAD, VAD, or MIXED dementia.

We first checked the age parameter, as it was significantly different between groups (see Table 1) and positively correlated with BACE1 activity (r: 0.269, *p* < 0.001). To abrogate the potential effect of age on BACE1 outcomes, we performed a further comparison after the exclusion of younger controls; the resulting sub-sample (n. 476 subjects) included groups with similar age (ANOVA *p*:0.20). As reported in Table 2, the differences between Controls and the other groups of patients were substantially unchanged. Of note, similar results were obtained when we compared younger controls with the two most homogeneous and numerous subsets of Other Dementia (i.e. Frontotemporal Dementia, n = 13; Lewy Body disease /Parkinson’s dementia, n = 13) (supplementary Table S1).

**Table 2:** Levels of serum BACE1 activity in controls, LOAD, VAD, MIXED, and other dementias after exclusion of younger controls.

Characteristics	Controls (n:82)	Load (n:175)	VAD (n:40)	Mixed (n:123)	Other dementias (n:56)
Age (years)	79 ± 4	78 ± 6	79 ± 7	80 ± 5	77 ± 6
BACE1 (kU/L)^#^	16 (13–20)	21 (16–27)^a^	23 (18–32)^a^	19 (17–26)^a^	17 (15–20)^b,c,d^

Continuous variables are expressed as mean ± SEM or median.

*Post-hoc test:* ^a^*p* < 0.05 versus controls; ^b^*p* < 0.05 versus LOAD; ^c^*p* < 0.05 versus VAD; ^d^*p* < 0.05 versus MIXED.

The potential confounding effect of gender on BACE1 was also evaluated due to the significant difference in gender across the sample groups (Table 1). In line with our previous analysis^16^, BACE1 was significantly higher in women (women: 19.2 kU/L vs men, 16.9 kU/L; *p* < 0.01). Despite this gender gap, the difference in BACE1 activity between Controls and LOAD, VAD or MIXED dementia was similar in women and men (Supplementary Table S2). As confirmation of these results, multivariable logistic regression analysis showed that higher BACE1 activity (IV quartile) was associated with the diagnosis of LOAD, VAD, and MIXED dementia after adjustment for age and gender (Supplementary Table S3).

At variance of age and gender, comorbidities and smoking did not affect BACE1 levels, and thus were not examined as potential confounders (data not shown).

### Diagnostic accuracy evaluation

**Figure 2:**
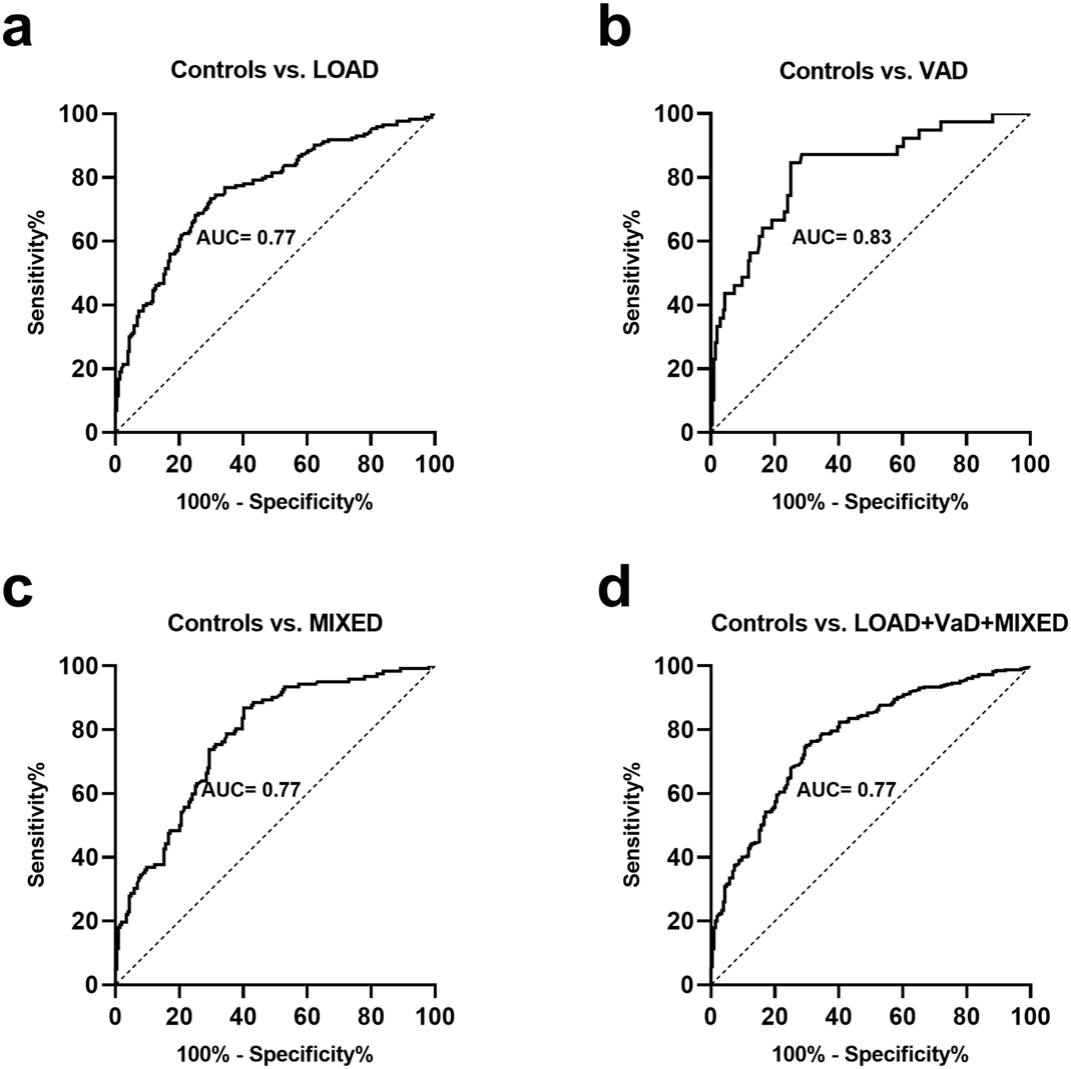
Receiver operating characteristic (ROC) curves for BACE1 activity for the diagnosis of LOAD, VaD, Mixed dementia, and the grouped LOAD + VaD + MIXED dementia. The calculated AUC values are 0.772 (Panel a, sensitivity/specificity: 73/70%) for LOAD, 0.831 (Panel b, sensitivity/specificity: 85/75%) for VAD, 0.771 (Panel c, sensitivity/specificity: 74/71%) for MIXED dementia, and 0.773 (Panel d, sensitivity/specificity: 75/70%) for the group including the 3 diseases all together.

Finally, the diagnostic accuracy of BACE1 was evaluated by ROC curves and the area under ROC curves (AUC) calculation (Fig. 2) using the best compromises between sensitivity and specificity for the diagnosis of LOAD, VAD and MIXED dementia, with a cut-off of BACE1 activity of 16.9, 18.0, and 17.00, respectively. AUC values were 0.772 (Fig. 2a, sensitivity/specificity: 73/70%) for LOAD, 0.831 (Fig. 2b, sensitivity/specificity: 85/75%) for VAD, 0.771 (Fig. 2c, sensitivity/specificity: 74/71%) for MIXED dementia, and 0.773 (Fig. 2d, sensitivity/specificity: 75/70%) for the group including the 3 diseases all together (Supplementary Table S4). We also calculated the positive and negative predictive values, which were 67% and 75% for LOAD, 40% and 96% for VAD, and 60% and 81% for MIXED dementia, respectively.

## Discussion

The present study was conceived as the logical continuation of our previous findings showing that BACE1 serum activity is increased in LOAD compared to Controls^17^. To this aim, we extended the investigation to a much larger population sample (n. 598), including not only a higher number of Controls and LOAD, but also other types of dementia.

Our efforts led us not only to confirm our previous results, but also to novel findings that might add to the current knowledge on the relationship between BACE and dementia. As a matter of fact, we showed that the increase in BACE1 serum activity is not a specific feature of LOAD, but also occurs in VAD and MIXED dementia. On the contrary, patients with other forms of dementia (such as Lewy body disease, frontotemporal, etc.) did not exhibit a significant alteration in BACE1 activity. Last but not least, BACE1 showed to perform even better in discriminating VAD from Controls when compared to MIXED dementia and LOAD (diagnostic accuracy values: 0.831, 0.772, 0.771, respectively).

Our data might appear contradictory at first glance. Indeed, BACE1 is classically ascribed as the key enzyme of amyloidogenic pathway that drives LOAD and not VAD pathogenesis. BACE1 is indispensable for the generation of Aβ since germline deletion of BACE1 gene abolishes Aβ deposition, preventing the subsequent development of amyloid-associated pathologies^19–21^. Moreover, several lines of evidence show that both protein levels and BACE1 activity are elevated in the brain regions affected by AD^22,23^. Although it might be challenging to establish from post-mortem tissue whether a specific change is a late or early event or an epiphenomenon in disease pathogenesis^24^, Cole et al. showed that BACE1 elevation was correlated with amyloid pathology in mouse models both in the absence (model: Tg2576) and presence (5XFAD) of significant neuronal loss^24,25^. In agreement with an early involvement of BACE1 in LOAD, enzyme activity was found to be higher in CSF and plasma of MCI converters compared to non-converters^16,18,26^.

On the other hand, there is a wealth of convergent data (“in vitro” and “in vivo” models) clearly suggesting that a vascular insult up-regulates BACE1^13,24,27,28^. Owing this data, the observed elevation of BACE1 in VAD and MIXED dementia is not surprising, but rather expected. BACE1 is currently described as a stress-response protein, sensitive to factors that can tackle energy metabolism in the brain^24,27^. These perturbing factors are often a direct consequence of cerebral micro/macro infarctions, particularly of chronic/acute cerebral hypo-perfusion. It is well known that this phenomenon gives rise to several interconnected abnormalities including hypoxia, inflammation, glucose/lipid dyshomeostasis and oxidative stress, which are all putative modulators of BACE1 expression and activity. In this frame, an emblematic example is the effect of hypoxia on BACE1 expression, that may occur by release of ROS from dysfunctional mitochondria and by activation of Hypoxia-inducible factor 1 (HIF-1), the master regulator of oxygen homeostasis^13,28,29^. The induced elevation in BACE1 may in turn affect vascular integrity and function, due to the well-recognized vasoactive proprieties of Aβ^24^.

We are aware that our cross-sectional findings do not allow to precisely define the role of BACE1 in dementia pathogenesis. However, our data led us to hypothesize that BACE1 might have a role in dementia that is far beyond its significance as possible biomarker. Based on our result, we propose that BACE1 might represent an important mechanistic link between VAD and LOAD. The overlap in symptomatology, pathological traits and risk factors has led some authors to propose that VAD and LOAD are similar diseases^30^. Others are more cautious about the idea of an equivalence between these two diseases, and suggest that vascular abnormalities might be the earliest and strongest pathologic factor, thus preceding Aβ formation/deposition^4,31^. This has been elegantly shown by Iturria-Medina et al. through the reconstruction of LOAD-abnormality trajectories by integrating brain imaging biomarkers and blood/CSF biological descriptors^4^.

**Figure 3:**
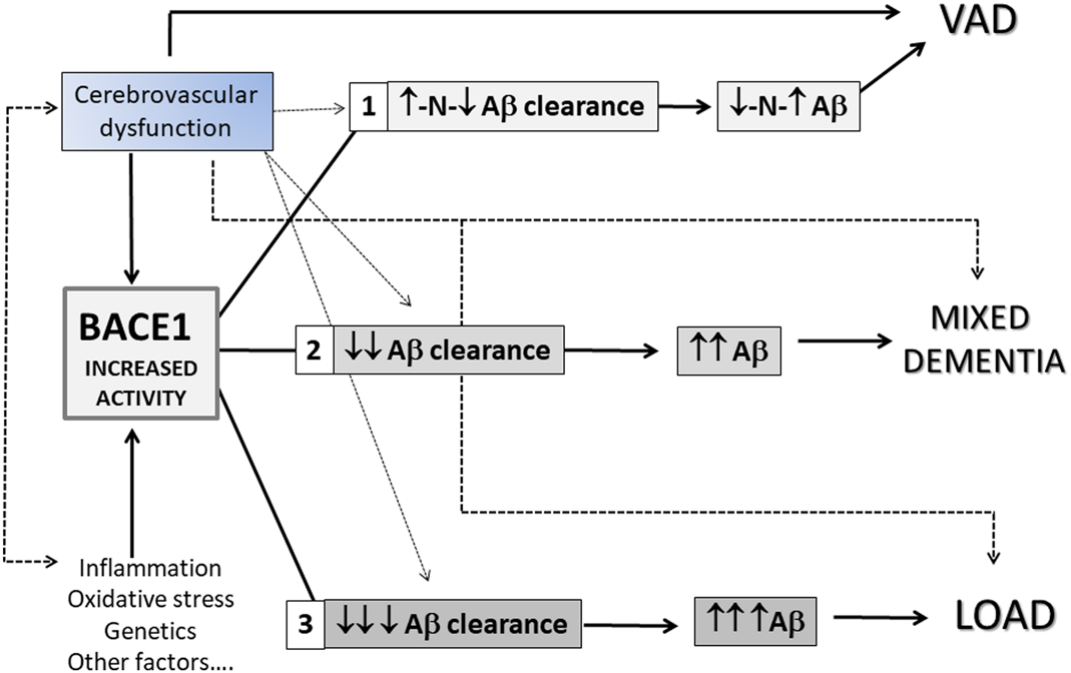
Potential impact of increased BACE1 activity in the pathogenesis of VaD, MIXED and LOAD. Cerebrovascular dysfunctions and/or other endogen/exogenous factors (e.g. oxidative stress, genetic predisposition, etc.) may cause increase in BACE1 activity leading to an increase in Aβ formation. This, in turn, can be accompanied by different levels of Aβ clearance efficiency and, as a consequence, by different levels of Aβ brain accumulation. The scenarios that can take place from different combination of increased BACE1/Aβ clearance effectiveness might predispose elderly individuals to different type of dementia: 1) VaD: partially impaired or unaffected Aβ clearance – mild increase/unchanged levels of Aβ 2) MIXED: partially impaired Aβ clearance—moderate to high levels of Aβ 3) LOAD: impaired Aβ clearance- high levels of Aβ.

Thus, by combining data pointing to a precocious alteration of BACE1 in LOAD^18,24,25^ with data of multifactorial-driven analysis^4^ and our current results, it is tempting to speculate a temporal cascade of events in LOAD (Fig. 3) in which vascular and other abnormalities induce BACE1 alteration. This, in presence of a concomitant defective Aβ clearance, would be followed by deposition of Aβ and neurodegeneration. Certainly, other factors might modulate all these pathogenetic steps. The fact remains that 1) a significant increase in BACE 1 activity seems to be a common phenomenon in LOAD and VAD; 2) the increase in BACE1 serum activity is absolutely comparable in LOAD and VAD, suggesting the possibility that other factors downstream Beta-amyloid production might direct brain pathology toward LOAD rather than VAD.

However, it is noteworthy that our diagnoses were not based on biomarkers; as a consequence, it cannot be excluded that AD pathology might be present in patients with probable VAD (it was expected instead that AD pathology would be present in MIXED dementia). With regards to this crucial point, it has to be emphasized that NINDS-AIREN criteria for probable VAD have a low sensitivity (about 20–60%) but a high specificity (about 90–99%) as reported by clinical and neuropathological studies^32,33^. Thus, while we might have missed some VAD diagnosis, the number of false positive would be very low. To confirm this, at autopsy a small percentage (10–15%) of demented patients with pure vascular damage and no AD pathology are found^34–36^. In this light, it appears unlikely that serum BACE1 is high in VAD as much as in LOAD just as a consequence of a low diagnostic specificity. Rather, the finding of elevated BACE1 levels in both LOAD and VAD (but not in other types of dementia) might have alternative explanations: 1. BACE-1 dysregulation together with AD pathology accumulation might be the cornerstone of both LOAD (no/minimal cerebrovascular disease) and VAD (significant cerebrovascular disease); at this point, the real existence of pure VAD from the nosological point of view could be at least questionable. This would be in line with the finding that all of patients with signs of cerebrovascular pathology at autopsy also had some concomitant neurodegenerative disease, particularly AD^37^. 2. On the other hand, since about 50% of AD patients also have cerebrovascular disease at autopsy^38^, the theoretical possibility exists that high BACE-1 serum levels might be actually driven by VAD and not by LOAD (shift of perspective).

Finally, we should also acknowledge two other important limitations of the study. First, the study was cross-sectional, thereby precluding our ability to establish any cause/effect relationship between BACE1 and cognitive impairment/dementia. Second, we cannot exclude that biases or unmeasured confounders might have also a role in the development of the pathologies. However, we took in consideration several potential confounders (age, gender, hypertension, diabetes, CVD, etc.) and the observed increase in BACE1 activity was independent from those factors.

In conclusion, we have demonstrated for the first time that BACE1 serum activity is increased in VAD and MIXED dementia as much as in LOAD, compared with healthy Controls; this increase is significant and is independent of possible measured confounders. On the contrary, BACE1 activity was not increased in other forms of dementia. Our findings further underline the possible shared characteristics between LOAD and VAD, highlighting the potential role of BACE1in the early pathogenesis of these two common types of dementia.

## Material and methods

### Subjects

A total of 598 older subjects referring to the Day Service for Cognitive Decline of University of Ferrara, or the Casa Sollievo della Sofferenza, San Giovanni Rotondo (Italy) were enrolled into the present study. The study sample included:

- 175 patients with mild to moderate probable LOAD determined by the National Institute on Aging–Alzheimer’s Association (NIA-AA) workgroups criteria^39^. Mini Mental State Examination (MMSE) range: 18–23; Clinical Dementia Rating (CDR) range: 1–2;
- 40 patients with probable VAD, according to National Institute of Neurological Disorders and Stroke and Association Internationale pour la Recherché et l'Enseignement en Neurosciences (NINDS-AIREN) criteria^33^. MMSE range: 16–23; CDR range: 1–2
- 123 patients with MIXED dementia; in these patients a definite diagnosis of LOAD or VAD was not possible since they presented both the characteristics VAD (e.g. significant vascular disease, focal neurological signs) and LOAD (e.g. memory impairment, type of progression). MMSE range: 16–23; CDR range: 1–2.
- 56 patients with other dementias (13 Lewy Body disease/Parkinson’s dementia, 13 frontotemporal dementia, 8 condition related to psychiatric conditions, 5 neoplasm/metastasis, 2 hydrocephalus, 2 Fahr’s syndrome, 2 alcohol related, 1 post syphilis, 1 hypothyroidism, 9 not defined). MMSE range: 18–25; CDR range: 1–2
- 204 healthy individuals (Controls) without any evidence of dementia and without functional disabilities attributable to cognitive impairment. MMSE range: 26–30.

Of note, 131 Controls and 115 LOAD were already examined in a previous study^17^.

There was no evidence of acute illnesses at the time of clinical observation and blood sampling. No subject was taking Nonsteroidal anti-inflammatory drug (NSAIDS), antibiotics, or steroids at the time of recruitment.

General and neuropsychological examination including MMSE and items geriatric depression scale (GDS), CDR, and MMSE was carried as previously described^40^. Functional status was also evaluated though basic activities of daily living (BADL) and instrumental activity of daily living (IADL). Personal data and medical history (e.g. hypertension, coronary heart disease, stroke, diabetes, chronic obstructive pulmonary disease) were collected by trained personnel^41^. Clinical chemistry analyses were routinely performed to exclude causes of secondary cognitive impairment including serum B-12 vitamin and folate, liver, kidney and thyroid function tests, blood cell count, and arterial oxygen saturation. The study was carried out in accordance with the guidelines provided by Declaration of Helsinki (World Medical Association, https://www.wma.net) and it was approved by the Local Ethic Committee of "Casa Sollievo della Sofferenza", San Giovanni Rotondo (protocol n. 3,877/DS) and Local Ethic Committee of "Azienda Arcispedale S. Anna", Ferrara (protocol n. 170,579). Signed informed consent, which was written in compliance with local and national ethical guidelines, was obtained from each patient prior to the inclusion into the study.

### BACE1 activity assay

Peripheral blood samples were collected by venipuncture into VACUTAINER tubes without anticoagulant after overnight fasting. After 30 min of incubation at room temperature, the blood samples were centrifuged at 4,650×*g* for 20 min and sera were collected and stored in single-use aliquots at − 80 °C until analysis.

The assay was carried out as previously described^17^. Briefly, 100 μL of substrate (i.e. H–K(Dabsyl)SEVNLDAEFRC(MAL-LY)-NH_2_ which was synthetized as described in^17^), at a final concentration of 30 μM in the assay, was added to the wells of a black flat-bottom microplate.

After pre-incubation for 10 min at 37 °C, the reaction was started by the addition of 5 μL of sample (wild-type enzyme or undiluted serum) in triplicate. The fluorescence was read every 30 s for 20 min using excitation and emission wavelengths of 430 nm and 520 nm, respectively, in a Tecan Infinite M200 (TECAN GROUP, Switzerland) microplate reader. The reaction rates were converted from relative fluorescence units (RFU) per minute to enzyme units (U) by interpolation with a standard curve constructed using known concentrations of the wild-type enzyme.

### Statistical analysis

Continuous variables were expressed as mean (standard deviation—SD) or median (interquartile range) when they were normally and non-normally distributed, respectively. Means were compared by t-test or ANOVA (with Sidak post-hoc test for multiple comparison); medians were compared by Kruskal–Wallis/Mann–Whitney U test. Proportion were compared by the χ2 test. Correlations were analyzed by Pearson’s and Spearman’s test according to their normal/non-normal distribution. Multivariable logistic regression analysis (using the highest quartile value as cut-off) was performed to determine the impact of covariates on the relationship between the variables of interest. A receiver operating characteristic (ROC) curve was performed to evaluate the diagnostic accuracy of the examined biomarker in discriminating controls from cases. Analyses were performed by SPSS for Windows statistical package, version 13.0.

## Supplementary information

Supplementary file1
