# Supplementary material for: Increased blood BACE1 activity as a potential common pathogenic factor of vascular dementia and late onset Alzheimer's disease
Source: Sci Rep. 2020 Sep 11;10:14980. doi: 10.1038/s41598-020-72168-3 (PMC7486910; doi:10.1038/s41598-020-72168-3)
Supplement: Supplementary file 1 — Supplementary file1 [file 41598_2020_72168_MOESM1_ESM.pdf]

Increased blood BACE1 activity as a potential common pathogenic factor of vascular dementia and late onset Alzheimer's disease

Giovanni Zuliani, PhD, Alessandro Trentini, PhD, Valentina Rosta, MS, Remo Guerrini, PhD, Salvatore Pacifico, PhD, Stefania Bonazzi, MD, Anna Guiotto, MS, Angelina Passaro, MD, Davide Seripa, PhD, Giuseppe Valacchi, PhD , Carlo Cervellati, PhD

Table S1. Levels of serum BACE1 activity in controls, Frontotemporal Dementias, Lewy Body disease/Parkinson's dementia, and other dementias after exclusion of younger controls.

Characteristics	CONTROLS (n:82)	Frontotemporal Dementia (n:13)	Lewy Body disease /Parkinson's dementia (n:13)	OTHER DEMENTIAs* (n:30)
**Age (years)**	79 ± 4	74 ± 6 [a,b]	78 ± 5	78 ± 5
**BACE1 (kU/L)**	16 (13-20)	15 (13-17)	19 (12-20)	18 (15-23)

Continuous variables are expressed as mean ± SEM or median
Post-hoc test: [a] $p < 0.05$ vs controls; [b] $p < 0.05$ vs other dementias
*The group includes: 8 condition related to psychiatric conditions, 5 neoplasm/metastasis, 2 hydrocephalus, 2 Fahr's syndrome, 2 alcohol related, 1 post syphilis, 1 hypothyroidism, 9 not defined

Table S2. Levels of serum BACE1 activity in controls, LOAD, VAD, MIXED, and other dementias in women and men.

	CONTROLS	LOAD	VAD	MIXED	OTHER DEMENTIAs
- **WOMEN**					
**BACE1 (kU/L)** [#]	16 (13-20)	21 (16-27) [a]	23 (19-31) [a]	21 (17-29) [a]	17 (15-21) [c,d]
- **MEN**					
**BACE1 (kU/L)** [#]	14 (12-17)	22 (17-26) [a]	22 (17-26) [a]	18 (16-24) [a]	18 (14-20)

Continuous variables are expressed as mean ± SEM or median
Post-hoc test: [a] $p < 0.05$ vs controls; [c] $p < 0.05$ vs VAD; [d] $p < 0.05$ vs MIXED

Table S3. Odds Ratios (95%C.I.) for diagnosis of LOAD, VAD or MIXED dementia in subjects with high levels (IV quartile, cut-off: 18.3 kU/L) of serum BACE1 activity.

		**Adjusted for age and gender**
**LOAD**	5.91 (3.78-9.22) *	5.25 (3.22-8.56) *
**VAD**	8.64 (3.94-16.92) *	5.69 (2.37-11.65) *
**MIXED**	4.40 (2.74-7.18) *	5.25 (3.22-8.56) *

*$p<0.001$

Table S4. Area under curve (AUC), sensitivity and specificity for ROC curves calculated at the optimal cutoff, of serum BACE1 to discriminate Controls from LOAD, VAD or MIXED

	**Cut-off (kU/L)**	**SE (%)**	**SP (%)**	**p value**	**AUC 95% CI**
**CONTROLS vs LOAD**	>16.9	73	70	<0.0001	0.772 (0.711-0809)
**CONTROLS vs VAD**	>18.0	85	75	<0.0001	0.831 (0.748-0.897)
**CONTROLS vs MIXED**	>17.0	74	71	<0.0001	0.771 (0.723-0.825)
**CONTROLS vs LOAD+VAD+MIXED**	>16.9	75	70	<0.0001	0.773 (0.732-0.813)

Abbreviations: SE, sensitivity; SP, specificity; AUC, area under the curve; CI, Confidence interval
